# Impact of Policies in Nutrition and Physical Activity on Diabetes and Its Risk Factors in the 28 Member States of the European Union

**DOI:** 10.3390/nu13103439

**Published:** 2021-09-28

**Authors:** Szabolcs Lovas, Nour Mahrouseh, Olaniyan Simisola Bolaji, Noel Johny Nellamkuzhi, Carlos Alexandre Soares Andrade, Diana Wangeshi Njuguna, Orsolya Varga

**Affiliations:** 1 Department of Public Health and Epidemiology, Faculty of Medicine, University of Debrecen, 26 Kassai Street, 4028 Debrecen, Hungary; lovas.szabolcs@med.unideb.hu (S.L.); nour.mahrouseh@med.unideb.hu (N.M.); soares.andrade@med.unideb.hu (C.A.S.A.); diana.njuguna@med.unideb.hu (D.W.N.); 2 Doctoral School of Health Sciences, University of Debrecen, 4032 Debrecen, Hungary; 3 Faculty of Medicine, University of Debrecen, 4032 Debrecen, Hungary; bolaniyan61@gmail.com (O.S.B.); noelnellamkuzhi@gmail.com (N.J.N.); 4 Eötvös Loránd Research Network, 1052 Budapest, Hungary

**Keywords:** policy impact, NOURISHING food policy database, MOVING physical activity policy database, correlation analysis, diabetes mellitus burden, European Union

## Abstract

Since healthy eating and physically active lifestyles can reduce diabetes mellitus (DM) risk, these are often addressed by population-based interventions aiming to prevent DM. Our study examined the impact of nutritional and physical activity policies, national diabetes plans and national diabetes registers contribute to lower prevalence of DM in individuals in the member states of the European Union (EU), taking into account the demographic and socioeconomic status as well as lifestyle choices. Datasets on policy actions, plans and registers were retrieved from the World Cancer Research Fund International’s NOURISHING and MOVING policy databases and the European Coalition for Diabetes report. Individual-based data on DM, socioeconomic status and healthy behavior indicators were obtained via the European Health Interview Survey, 2014. Our results showed variation in types and numbers of implemented policies within the member states, additionally, the higher number of these actions were not associated with lower DM prevalence. Only weak correlation between the prevalence of DM and preventive policies was found. Thus, undoubtedly policies have an impact on reducing the prevalence of DM, its increasing burden could not be reversed which underlines the need for applying a network of preventive policies.

## 1. Introduction

### 1.1. Diabetes and Increasing Disease Burden

Non-communicable diseases (NCDs), such as diabetes mellitus (DM), cannot be transmitted directly from one person to another. NCDs are implicated to cause 71% of all deaths, globally [1]. DM falls within the top four predominating NCDs across the world, and its significance is amplified in the European Union (EU) [2]. Type 2 diabetes mellitus (T2DM) is accounts for 90% of the total diabetes cases [3]. NCDs including T2DM are largely preventable diseases due to their lifestyle-related risk factors including smoking, alcohol consumption, inappropriate diets and low level of physical activity [4,5,6]. In most member states of the EU, there is a falling or stagnating trend of disability-adjusted life year (DALY) rates, which is a composite measure of number of years lost due to disability and early death to diseases such as neoplasms, cardiovascular and respiratory diseases, which occurred during the last decade [7,8]. The rising trend of DM is rather exceptional among the NCDs since it increased from 956 DALYs per 100,000 in 2010 to 1099 in 2019 in the EU member states [9,10].

Although the economic impact of diabetes is among the less studied fields, the cost-of-illness studies consistently report increasing diabetes-related expenditure due to its increasing prevalence, treatment and societal costs [11,12,13]. Individuals having DM are more likely to use the healthcare resources which results in higher direct costs [14]. Type 2 diabetes mellitus also increases the labor-force exit by 30%, and the probability of having disability benefit is more than twofold [15,16]. Health expenditures linked to diabetes pose a significant public health challenge which negatively affects the sustainability of healthcare systems [17,18]. Effective prevention programs targeting chronic NCDs can save significant costs [19].

### 1.2. International Initiatives to Manage DM in the European Union

In 1989, 45 European states held at St. Vincent in Italy to set a common strategy for DM which is called St Vincent Declaration [20]. In harmony with this declaration, several EU member states have launched national plans addressing diabetes exclusively or incorporated into wider NCD strategies. Although the initial impetus was broken, due to the effort of the International Diabetes Federation—European Region (IDF Europe) and the Federation of European Nurses in Diabetes (FEND), the European Parliament adopted a Written Declaration on Diabetes in 2006. The declaration required the Commission and Council to give priorities to diabetes by facilitating the introduction of national diabetes plans and by developing an EU diabetes strategy in the form of an EU Council Recommendation on Diabetes Prevention, Diagnosis and Control.

The EU Ministers of Health adopted the Conclusions on the Promotion of Healthy Lifestyles and Prevention of Type 2 Diabetes in 2006 [21]. This document states that member states are to develop and implement national diabetes plans, and among others to ameliorate the collection and reporting of epidemiological and economic data of DM, as well as to apply a multi-sectoral and multidisciplinary approach to DM management. Furthermore, the Council called upon the European Commission to facilitate and support networking and diabetes research in Europe.

In 2012, the European Parliament adopted a resolution calling on the Commission to develop and implement a targeted EU diabetes strategy, including key dimensions of prevention, diagnosis, treatment, education and research [22]. Furthermore, calls were made for standardized criteria and methods for diabetes data collection and for the coordination, collection, recording, monitoring and management of comprehensive epidemiological data on DM and economic data on the direct and indirect costs of DM prevention and treatment in collaboration with the member states [23].

### 1.3. Regulation of Policies Related to Prevention of NCDs in the EU and Its Member States

Despite efforts to highlight DM, today its prevention is mainly integrated into population-based initiatives to prevent NCDs as a group. A recent joint paper entitled Towards an EU-Strategic Framework for the Prevention of Non-communicable Diseases has concluded need for establishing EU strategic framework to prevent NCDs [24].

Currently, in the EU, the individual member states and the EU have the legislative powers and responsibility to create rules and regulation to prevent NCDs. The Treaty on the Functioning of the European Union (TFEU) underlines the utmost importance of health by stating that ‘a high level of human health protection shall be ensured in the definition and implementation of all Community policies and activities’ (Article 168 (1) of the TFEU) [25]. However, the responsibility for health is not shared equally between the EU and its member states: the primary responsibility for health protection and other functions of healthcare systems with the member states, the EU itself has an explicit competence in well-defined areas of public health (Article 168). Article 168 TFEU does not provide legal basis allowing EU-wide binding harmonization of national laws to prevent NCDs. Since Article 168 TFEU provides opportunity to the EU to produce non-binding, supportive actions and mandates in the field of health, several non-binding policies to prevent NCDs including guidelines, green papers or international commitments address obesity, physical inactivity and unhealthy diets such as the Joint action on Chronic Diseases (JA-CHRODIS), Joint action on Nutrition and Physical Activity (JA-NPA), European Charter on Counteracting Obesity (2006), the European Framework on Physical Activity (2007) and the European Action Plan for Food and Nutrition Policy 2007–2012 were published [26,27,28,29].

However, Article 114, which deals with the harmonization of the internal market, plays an important role in the regulation of public health. For example, Article 114 (3) was taken as a base for legislation of the Tobacco Product Directive (Directive 2014/40/EU) or the Regulation of the European Parliament and of the Council on the provision of food information to consumers (1169/2011/EU) [30,31].

### 1.4. Objectives

This study examines: (1) the impact of nutritional and physical activity policies, national diabetes plans and national diabetes registers on prevalence of diabetes in the member states of the EU, and (2) the association between demographic and socioeconomic status as well as the lifestyle choices, taking into account available policies.

The overall hypothesis of the study is that member states of the EU with one or more implemented policies addressing healthy diet, physical activity, and with a national diabetes plan and/or diabetes register, have lower DM prevalence.

## 2. Materials and Methods

### 2.1. Data Sources

Four data sources were used in this study: the World Cancer Research Fund International’s (WCRFINT) NOURISHING and MOVING policy databases [32], the European Coalition for Diabetes report “Diabetes in Europe policy puzzle: the state we are in” [33] and The European Health Interview Survey (EHIS) 2014.

### 2.2. Policies, National Plans and National Diabetes Registers

NOURISHING and MOVING policy databases consist of information on implemented nutrition and physical activity policies, worldwide. The policies were collected by the World Cancer Research Fund (WCRF) through questionnaires [34]. All policies included in the databases have been verified by national governmental experts. Policies in this context included regulations, directives, strategies, campaigns or other legislative tools targeted nutrition or physical activity. Policies are grouped into three levels, policy categories, policy areas, and sub-policy area; policy categories and policy areas are presented in Table 1 and Table 2. Policy areas according to the NOURISHING database covers wide range of policies such as regulating food advertisement, labeling and health claims, initiating public health promotional and educational campaigns to the public communities and in health care settings, providing affordable healthy food using economic tools and incentives not only in specific setting but also to have healthy retail service environment, and supply chain. Policy areas from the MOVING database are focused on initiatives promoting physical activity in schools, workplace and other settings, promoting and educating aspects of active living and sport training through public campaigns and in health care and creating enabling environment through infrastructure and transport planning that support active societies. See Supplementary Materials Table S1 for further clarifications on policy areas and sub-policy areas. For the analysis, policies implemented by the 28 EU member states, targeting adult populations partially or exclusively that were in force in 2014, were retrieved.

National diabetes plans were introduced in many EU countries to overcome the rise in DM prevalence in past decades, which included comprehensive plans based on broad government commitment from prevention action areas to treatment strategies, addressing diabetes exclusively or as part of a plan for NCDs.

National diabetes registry systems are databases where the clinical course of patients with type 1 diabetes mellitus and/or T2DM could be monitored and based on the collected data, targeted preventive measures can be applied. Databases excluding T2DM were not considered during the analysis. National diabetes plans and national diabetes registers, effective in 2014, were extracted from the fourth edition of “Diabetes in Europe policy puzzle: the state we are in”. This report is based on a standardized questionnaire distributed to and collected information from specialists and officials to obtain the most accurate information about the country profile in managing diabetes [33].

### 2.3. Study Sample

Data from Eurostat, European Health Interview Survey (EHIS) 2014 were used for the 28 member states of the European Union, including the United Kingdom (UK). EHIS is an integral part of the European Commission health related activities providing cross-national data on health status, health care and health determinants. EHIS is collected every 5 years where participants are at least 15 years old and live in private households [35]. The EHIS data collected are comparable and carry relevant information for European health policy surveillance allowing to establish evidence-based policy decisions in the field of public health [36,37].

Our data consisted of 304,168 observations. Presence of diabetes was considered based on a self-reported question: “During the past 12 months, have you had diabetes?” Respondents who answered “Yes” to this question were considered in the group as individuals having diabetes.

Demographic, socioeconomic and lifestyle characteristics assessed were sex, age (15–44, 45–64, 65 and above), degree of urbanization (cities, towns and suburbs and rural areas), educational attainment (less than primary and primary education, secondary education and higher education), labor status (employed, unemployed and others e.g., retired student), net monthly equalized income of the household the respondent belongs to (between 1st quintile and 2nd quintile, between 2nd quintile and 4th quintile and between 4th quintile and 5th quintile), frequency of eating fruits and frequency of eating vegetables per week (one and more per day, 1 to 6 times a week and less than once a week and never). Definitions of the variables are provided in the Supplementary Materials file S1, Table S2.

### 2.4. Statistical Analysis

The distribution of the variables was described and compared for diabetic and non-diabetic respondents. Estimated prevalence of diabetes in 2014 was calculated for all the member states of the EU, sample weight was used to calculate prevalence.

Chi-square test was used to determine significant predictors associated with diabetes. Point-biserial correlation analysis of the numbers of policies of first level of nutritional and physical activity and the availability of national plan and/or register in each member state and the prevalence of diabetes for all the study sample and stratified by age and sex was calculated. Point-biserial Correlation Coefficient measures the strength of association ranging from −1 to +1, where −1 indicates a negative association, +1 indicates a positive association and 0 indicates no association [38]. In order to consider the hierarchical structure in the data, a multi-level logistic regression model was applied for diabetes adjusting for individual and country-level variables. Country (place of residence) was considered as level-2 factor. The overall aim of multi-level logistic regression was to estimate the odds of occurring of an event, while taking the dependency of data into account. All statistical analyses were carried out in STATA version 16.0^®^.

## 3. Results

### 3.1. Overview of the Study Population

Table S3 in Supplementary Materials file 1 shows the distribution of the study sample by numbers and relative frequencies, comparing diabetic and nondiabetic respondents. Results of chi-square test showed, there are significant relationships between the prevalence of diabetes and the study variables sex, age, educational attainment, labor status, net monthly equalized income of the household the respondent belongs to, BMI, frequency of fruit and vegetable consumption.

Among the participants, 22,566 were reported with DM. In the EU, the estimated prevalence of DM was 6.94% (6.82–7.06) in 2014. Estimated prevalence percentage in France 9.98% (9.45–10.54), Portugal 9.33% (8.76–9.93) and Greece 9.24% (8.56–9.97) reported the highest values. The lowest prevalence percentage values were reported in Lithuania 4.41% (3.92–4.97), Denmark 4.62% (4.14–5.15) and Ireland 4.63% (4.22–5.08).

### 3.2. Overview of Nutrition and Physical Activity Policies, National Diabetes Plans and National Diabetes Registers in the EU Member States

Table 1 and Table 2 display the presence of nutrition and physical activity policies, national diabetes plans and national diabetes registers supplemented with the prevalence of diabetes and their 95% confidence interval in the 28 member states of the European Union in 2014.

In that year, most nutrition and physical activity policies implemented and in force were in the United Kingdom (34), meanwhile Malta (2), Slovakia (2), Luxembourg (2) and Lithuania (2) had the lowest number of applied policies according to the WCRFINT’s database. Nutrition label standards and regulations on the use of claims and implied claims on food was the only policy area introduced in all 28 member states. Policies addressing set incentives and rules to create a healthy retail and food service environment and give nutrition education and skills were the least frequently applied. The latter was not introduced in any member state in 2014.

In total, 17 out of the 28 member states had existing national diabetes plan and seven member states had operating national diabetes registry system in 2014.

### 3.3. Individual Level Correlation between National Policies, Diabetes Plans and Registers and Diabetes Prevalence

Results obtained from point biserial correlation analyses found food environment and food system policies had positive weak correlation with prevalence of diabetes, which showed significance for total population, males and age groups between 15 and 44 and 45 and 64. For age group of 65 and above, food environment policies and food systems policies showed a weak negative significant correlation with DM.

Behavior change communication policies had positive weak correlation with prevalence of DM, which showed significance for the total population and all sex and age groups except, for age group 65 and above, which revealed a weak negative significant correlation with DM.

Active society policies had only weak significant negative correlation with prevalence of DM in our total population group and its sex and age subgroups. 

Higher number of active environment policies had weak positive correlation with DM in our total sample, in males also, and age groups of 15–44 and 45–64, with significance. Higher number of active people policies demonstrated weak positive correlation with DM in the total population, in the male group and age group of 15–44. In addition, negative significant correlation with age group of 65 and above and active people policies was detected. Increased number of national diabetes plans and national diabetes registers had negative correlation with total population group, in females, and age groups of 15–44 and 45–64, see Table 3.

### 3.4. Country Level Correlation between National Policies, Diabetes Plans and Registers and Diabetes Prevalence

Variables which were found to be significant in the bivariate analysis of the study and of epidemiological interest were added to the multilevel logistic regression analysis model. Results of the multilevel logistic regression and the intraclass correlation coefficient obtained from multilevel logistic regression are reported in Table 4.

The variation attributable to countries as a grouping factor for diabetes after adjusting for individual, country level variables was small (ICC: 1.3%). The observed variability in prevalence of diseases between countries arose from demographic, socioeconomic and lifestyle characteristics, and not attributed to differences in availability and numbers of different preventive policies. None of the policy types had any significant association with the likelihood of having DM. Results of the multilevel analysis showed that females, living in less urbanized settings (suburban and rural), higher education (secondary and tertiary), middle and lower household income (between quintiles 2 and 4 and between quintiles 4 and 5), and fruit consumption 1–6 times per week significantly influenced the prevalence of the disease, with lower odds of being effected compared to their relative reference group. Unlike the participants in higher age groups (45–64 and 65 and above), labor status (unemployed and others) and higher ranges of BMI (25–29.9 and ≥30) were significantly increasing the probability of prevalence of the disease comparing to their relative reference group.

## 4. Discussion

### 4.1. Summary of Main Findings

In order to reduce the burden of DM, EU member states have either addressed DM directly by framing their actions through national disease plans and running diabetes registries, or indirectly through nutrition and physical activity policies that target the main risk factor for obesity. Although some relevant policies were implemented in all member states, our study results show large variations in their types and numbers. However, the higher number/any types of policies seem not to ensure a reduction in prevalence of DM, only a weak correlation between the prevalence of DM and preventive policies was detected. The differences in DM prevalence observed between member states are likely to be due to socio-economic differences and lifestyle characteristics of individuals in the EU, rather than to the policies implemented. 

### 4.2. Interpretation of Our Data

#### 4.2.1. Impact of Having Policies/Plans/Registries on Prevalence

Although our results show a considerable difference between the highest and lowest prevalence of DM (5.57%) across the member states, the analyses from the Global Burden of Disease 2019 show an upward trend until today in these countries [10]. National governments are increasingly required to implement policies and measures to prevent DM [39]. The two dominant strategies to prevent DM carried out by governments are to implement policies either promoting healthy eating or increasing physical activity [40]. There is an agreement in the literature that law has played a critical role in controlling chronic diseases and the behaviors that cause them [41,42]. The application of a systematic legal framework addresses a wide range of potential factors that changes DM environment can effectively help its prevention.

Considerable variation in the number of implemented nutritional policies was found in our study, ranging from 2 to 34; four countries had policies exclusively from the area of nutritional labelling. The more under-used categories of nutrition policies were the use of economic instruments for food affordability and restrictions on food advertising, reflecting the limited willingness of national governments to take regulatory action and their preference for educational strategies. This kind of legislative preference was already reported, e.g., by an analysis on maternal and child health policies from England and local policies for Southampton City [43]. Physical activity policies also show a wide variation across member states, ranging from 0 to 15; majority of countries were without implemented policies. Of all physical activity policies, the category of providing physical activity opportunities in the workplace was the least used, perhaps reflecting governments’ distance from the industrial sector. 

Similar to our findings from the EU, a large variety across public policies and interventions for DM in Latin America was observed by Kaselitz [44], underlining that the introduction of most policy actions is not evidence-based, and more research is needed to “determine their effectiveness, cost, and scalability”.

Results obtained from this study found no or weak correlation between DM prevalence and any category of nutritional/physical activity policies/existing diabetes plans/registries. Product labelling was the only legislative category applied in all the member states emphasizing the legislative role of the EU. In fact, labelling may have an impact on human behavior, such as promoting healthy food choice. The use of labelling system is widespread and well-known in the EU member states due to the obligatory national implementation of the Regulation (EU) No 1169/2011, however member states may apply additional labelling systems. Labelling is an important source of information, if such information is not embedded in context, it may provide limited help for interpretation. For example, health warnings are hardly used for ingredients (except for allergens) which is recommended for high sugar content [45] or nutrition labels on portion size selection [46].

Although economic instruments for food affordability and restrictions on food advertising were hardly applied in the member states, the so-called upstream dietary and multi-component interventions, including price changes, are found to be consistently effective in promoting healthy eating [47]. However, the effectiveness of the other interventions such as labelling or restrictions on the provision or marketing of unhealthy food are reported to have less effects and less certain long-term benefits [47]. Some EU countries (e.g., France, Hungary) have levied taxes on sugar-sweetened drinks, but the actual taxes have only increased the retail price by a relatively limited percentage. Significant behavior change is expected due to combined intervention of plain packaging, warning labels and a 20% tax on predicted sugar-sweetened beverages preferences [48].

The effectiveness of policies for promoting physical activity is also intensively studied. According to a recent systematic review, there is fairly strong evidence of the effectiveness of policies in certain areas, such as school settings for children and promoting walking and cycling. For a number of other categories, the evidence is mixed, with many studies suggesting effectiveness, while others have found only moderate or insufficient evidence. This seems to be the case for policies on children’s out-of-school settings, other settings or target groups, and policies on the built environment/active transport [49]. Such correlation between DM prevalence and school policies was not confirmed by our study likely due to the fact that EHIS dataset includes population above 15 years, exclusively. The fact that physical activity policies for children in a school setting are effective, but initiatives using a similar approach for adults in workplace settings are not [50], certainly needs to be considered by future research. This discrepancy highlights the fact that the effectiveness of policies depends on the age and other demographic features.

Populations are not homogeneous groups of people. In order to better understand the impact of policies, results were stratified by age and gender. Population structure influences the efficacy of nutritional and physical activity policies. For example, age and gender were found to be correlated with the potency of policy interventions managing NCDs [51]. The association between age groups and taxation policies was reported by a paper addressing SSB tax on obesity incidence in Portugal. The simulation study found that the biggest projected impact was expected in adolescents 10 to <18 years old [52]. While our work could not identify any subgroup that is consistently and clearly correlated with the impact of any nutritional and physical activity policies, further investigation is important. Cost-effective policy interventions may require policy refinements according to features of population subgroups. 

Regarding diabetes plans and registries, almost half of the member states had no diabetes plan and only 25% had national registries in 2014. The number of policies was not linked to the presence of diabetes plans and/or registers; as a prime example, the United Kingdom with the most implemented policies in the database had no national plan or nationwide diabetes registry in 2014. This discrepancy might be explained by NCDs taken approach (focusing on risk factors of the NCDs), instead of disease focus. However, the frequent lack of registries clearly indicate that DM is not in the center of public health policies in many member states, since population-based registries are considered as pillar of assessment of policy interventions [53].

#### 4.2.2. The Association between DM Prevalence and Demographic and Socioeconomic Status as Well as the Lifestyle Choices, Taking into Account Available Policies

Living in a country by itself can be a risk factor, for example the use of a low or high-risk chart of SCORE for cardiovascular risk levels depends on the country of residence [54]. In our analysis, the variation attributable to countries as a grouping factor for diabetes was small. The differences in disease prevalence observed between countries were due to demographic, socioeconomic and lifestyle characteristics, and not due to differences in the availability and number of different preventive measures. No policy type showed a significant association with the likelihood of DM. According to the results of multilevel analysis, the gender of participants as females, lower age groups, higher degree of urbanization, mid/low income and higher level of education were significantly influencing lower likelihood of prevalence of the disease. In addition, having higher BMI that considered overweight or obese significant effect on the probability of having diabetes, unlike some healthy eating habits as eating fruits 1 to 6 times per week which may significantly associate with lower burden of disease. 

To our best knowledge, the association between policies and prevalence of DM across EU countries has not been studied yet. This work has two important implications. One, the impact of socioeconomic policies on health status including DM cannot be overestimated. Policy makers addressing DM may achieve results via fighting against poverty, educational inclusion, better access to educational services, etc. The other implication is that marginalized policies cannot achieve the desired impact of DM burden reduction. 

Our analysis showed that no country fully covered the proposed categories by the WCRF of nutrition and physical activity policies. Forecast studies to assess policy impacts in DM prevention are increasingly used [55,56]. A simulation study reinforced the need for a network of policies to obtain the desired results. In that model, when all interventions were integrated, the population risk ratios for both obesity and T2DM decreased [57]. Isolated regulatory interventions targeting population nutrition may have a positive effect on intermediate outcomes, but this change does not reach clinically significant levels. e.g., policy effect on dietary intake that may lead to a reduction in the incidence of obesity or NCDs [58]. A systematic review by Sisnowski analyzed six different types of interventions and no evidence was found that the studied policies had the expected effect on risk factors and health outcomes [58]. Likewise, a review of systematic reviews found no evidence that any of the fragmented interventions examined had an effect on the prevalence of overweight, obesity or T2DM but intermediate achievements were provided [59]. 

In conclusion, as the only policy area that was actually regulated across member states was labelling, which is a consequence of the existence of an EU regulation, it implies the need to launch international treaties or other binding legislation. Involving international agencies to develop sound policies and control their implementation seems crucial [60,61,62]. 

### 4.3. Limitations

An important limitation of the study is the cross-sectional design which does not allow causality to be established. 

In our analysis, data from 2014 were used, which is the latest available dataset from EHIS during the manuscript preparation. The survey was based on self-reported questionnaires which may result in inaccuracy of the data and the conclusions drawn. Unfortunately, physical activity variables could not be assessed by the study since two countries, Netherlands and Belgium, did not provide data on those variables. Furthermore, policy databases may not cover the full spectrum of policies that were in effect in 2014; also, policies may need a longer time scale to achieve their full potential.

Although DM covers all diabetes subtypes with diverse pathophysiology, such diversity was not considered in the manuscript; the overall category of DM was used during the analysis. In fact, T2DM is responsible for by far the majority of cases of DM, nutritional and physical activity policies are geared towards risk factors for T2DM. Conclusions drawn from this study require careful implementation given the low number of policies.

## 5. Conclusions

Our work recommends that a higher implementation of nutritional and physical activity policies is not necessarily associated to lower prevalence of diabetes. In fact, some variability in nutritional and physical activity policies are present in the member states of the EU, intensively regulated fields are always due to efforts of EU legislation. Unquestionably, policies implemented until 2014 had some impact on reducing DM burden, but not enough to change such an upward trend. We believe that a matrix of policies is needed to manage diabetes burden, limiting the interventions to a few policy categories that are easier to implement for political and other reasons cannot be sufficient. Further studies are necessary to confirm the scale of policies’ impact on the burden of diabetes. 

## Figures and Tables

**Table 1:** Number of implemented government policy actions promoting healthy diets and reducing obesity and national diabetes plans in effect in the EU member states, 2014.

Member States of the European Union	Diabetes Burden		Nutritional Policies										National Diabetes Plan
			Categories										
			Food Environment						Food System	Behavior Change Communication			
			Policy Areas										
	Prevalence %	95% CI	Nutrition Label Standards and Regulations on the Use of Claims and Implied Claims on Food	Offer Healthy Food and Set Standards in Public Institutions and Other Specific Settings	Use Economic Tools to Address Food Affordability and Purchase Incentives	Restrict Food Advertising and Other Forms of Commercial Promotion	Improve Nutritional Quality of the Whole Food Supply	Set Incentives and Rules to Create a Healthy Retail and Food Service Environment	Harness Supply Chain and Actions across Sectors to Ensure Coherence with Health	Inform People about Food and Nutrition through Public Awareness	Nutrition Advice and Counselling in Healthcare Settings	Give Nutrition Education and Skills	
France	9.98	(9.45–10.54)	2	0	1	1	1	0	0	3	0	0	No
Portugal	9.33	(8.76–9.93)	2	0	0	0	1	0	0	1	0	0	Yes
Greece	9.24	(8.56–9.97)	2	0	0	0	1	0	0	1	0	0	Yes
Malta	8.25	(7.49–9.09)	2	0	0	0	0	0	0	0	0	0	No
Hungary	8.06	(7.37–8.81)	1	0	1	0	3	0	0	1	0	0	No
Finland	7.73	(7.07–8.44)	3	1	0	0	0	0	1	1	0	0	Yes
Czech Republic	7.67	(7.06–8.32)	3	0	0	0	1	0	0	0	0	0	Yes
Germany	7.17	(6.80–7.56)	2	1	0	0	0	0	0	1	1	0	No
Croatia	7.13	(6.47–7.86)	2	0	0	0	1	0	0	1	0	0	Yes
Slovakia	6.86	(6.25–7.53)	2	0	0	0	0	0	0	0	0	0	Yes
Slovenia	6.85	(6.21–7.56)	3	0	0	0	0	0	0	1	0	0	Yes
Spain	6.83	(6.47–7.22)	2	0	0	0	1	0	0	1	1	0	Yes
Italy	6.66	(6.34–6.98)	2	0	0	0	1	0	0	0	0	0	Yes
Poland	6.64	(6.30–6.98)	3	0	0	0	0	0	0	1	0	0	Yes
Bulgaria	6.37	(5.81–6.98)	2	0	0	0	1	0	0	1	0	0	Yes
Cyprus	6.06	(5.45–6.73)	2	0	0	0	0	0	0	1	0	0	No
United Kingdom	5.8	(5.47–6.14)	6	2	0	0	2	2	2	2	3	0	No
Luxembourg	5.57	(4.90–6.33)	2	0	0	0	0	0	0	0	0	0	No
Estonia	5.49	(4.91–6.14)	2	0	0	0	0	0	0	1	0	0	No
Netherlands	5.38	(4.90–5.91)	3	0	0	0	2	0	0	1	0	0	Yes
Belgium	5.34	(4.73–6.01)	3	0	0	1	1	0	0	0	0	0	No
Austria	4.93	(4.48–5.42)	2	0	0	0	2	0	0	1	0	0	Yes
Romania	4.79	(4.44–5.15)	2	0	0	0	0	0	0	1	0	0	Yes
Sweden	4.75	(4.21–5.37)	3	0	0	0	0	0	0	0	0	0	Yes
Latvia	4.66	(4.21–5.16)	1	1	1	0	0	0	0	1	0	0	No
Ireland	4.63	(4.22–5.08)	1	0	0	0	1	0	0	0	0	0	Yes
Denmark	4.62	(4.14–5.15)	4	0	0	0	1	0	0	1	0	0	Yes
Lithuania	4.41	(3.92–4.97)	2	0	0	0	0	0	0	0	0	0	No

(CI) confidence interval. Color gradient, from light to dark blue, is based on the number from lower to higher in each cell.

**Table 2:** Number of implemented government policy actions targeting physical activity and national diabetes register in operation in the EU member states, 2014.

Member States of the European Union	Diabetes Burden		Physical Activity Policies						National Diabetes Register
			Categories						
			Active Societies		Active Environments		Active People		
			Policy Areas						
	Prevalence %	95% CI	Make Opportunities and Initiatives that Promote Physical Activity in Schools, the Community and Sport and Recreation	Offer Physical Activity Opportunities in the Workplace and Training in Physical Activity Promotion across Multiple Professions	Visualize and Enact Structures and Surroundings Which Promote Physical Activity	Implement Transport Infrastructure and Opportunities That Support Active Societies	Normalize and Increase Physical Activity through Public Communication That Motivates and Builds Behavior Change Skills	Give Physical Activity Training, Assessment and Counselling in Healthcare Settings	
France	9.98	(9.45–10.54)	0	2	3	1	1	0	No
Portugal	9.33	(8.76–9.93)	3	1	2	1	0	0	Yes
Greece	9.24	(8.56–9.97)	0	0	0	0	0	0	No
Malta	8.25	(7.49–9.09)	0	0	0	0	0	0	Yes
Hungary	8.06	(7.37–8.81)	0	0	0	0	0	0	No
Finland	7.73	(7.07–8.44)	0	0	0	0	0	0	No
Czech Republic	7.67	(7.06–8.32)	0	0	0	0	0	0	No
Germany	7.17	(6.80–7.56)	1	1	2	5	0	0	No
Croatia	7.13	(6.47–7.86)	0	0	0	0	0	0	Yes
Slovakia	6.86	(6.25–7.53)	0	0	0	0	0	0	No
Slovenia	6.85	(6.21–7.56)	0	0	0	0	0	0	No
Spain	6.83	(6.47–7.22)	0	2	1	0	4	1	No
Italy	6.66	(6.34–6.98)	1	0	0	0	1	0	No
Poland	6.64	(6.30–6.98)	2	0	0	0	1	0	No
Bulgaria	6.37	(5.81–6.98)	0	0	0	0	0	0	No
Cyprus	6.06	(5.45–6.73)	0	0	0	0	0	0	Yes
United Kingdom	5.8	(5.47–6.14)	2	1	2	3	3	4	No
Luxembourg	5.57	(4.90–6.33)	0	0	0	0	0	0	No
Estonia	5.49	(4.91–6.14)	0	0	0	0	0	0	No
Netherlands	5.38	(4.90–5.91)	1	0	1	1	0	1	No
Belgium	5.34	(4.73–6.01)	0	0	0	0	0	0	No
Austria	4.93	(4.48–5.42)	0	0	0	0	0	0	No
Romania	4.79	(4.44–5.15)	0	0	0	0	0	0	No
Sweden	4.75	(4.21–5.37)	0	0	0	0	0	0	Yes
Latvia	4.66	(4.21–5.16)	1	0	1	0	0	0	Yes
Ireland	4.63	(4.22–5.08)	0	0	0	0	0	0	No
Denmark	4.62	(4.14–5.15)	0	0	0	0	0	0	Yes
Lithuania	4.41	(3.92–4.97)	0	0	0	0	0	0	No

(CI) confidence interval. Color gradient, from light to dark blue, is based on the number from lower to higher in each cell.

**Table 3:** Point biserial correlation coefficient between policies type and the prevalence of diabetes in 2014 in total population, and stratified by sex and age groups.

	Food Environment	Food System	Behavior Change Communication	Active Society	Active Environment	Active People	National Diabetes Policies and Registers
Total							
coefficients	0.009	0.009	0.017	0.035	0.018	0.011	−0.003
*p*-value	<0.001	<0.001	<0.001	<0.001	<0.001	<0.001	0.110
Females							
coefficients	−0.003	−0.004	0.007	0.032	0.004	0.002	−0.006
*p*-value	0.265	0.119	0.006	<0.001	0.116	0.375	0.021
Males							
coefficients	0.022	0.024	0.029	0.038	0.034	0.021	<0.001
*p*-value	<0.001	<0.001	<0.001	<0.001	<0.001	<0.001	0.946
Age between 15–44							
coefficients	0.023	0.008	0.035	0.017	0.028	0.006	−0.026
*p*-value	<0.001	0.006	<0.001	<0.001	<0.001	0.046	<0.001
Age between 45–64							
coefficients	0.008	0.005	0.015	0.029	0.014	0.002	−0.002
*p*-value	0.014	0.146	<0.001	<0.001	<0.001	0.433	0.577
Age 65 and above							
coefficients	−0.027	−0.025	−0.014	0.027	0.006	−0.015	0.006
*p*-value	<0.001	0.000	<0.001	<0.001	0.094	<0.001	0.104

**Table 4:** Association of diabetes mellitus and demographic, socioeconomic, lifestyle and number of policy types.

Variable	Odds Ratio	95% Confidence Interval	*p*-Value
ICC	0.013		
Food environment	0.98	(0.91–1.06)	0.688
Food system	1.06	(0.75–1.49)	0.753
Behavior change communication	1.11	(0.94–1.31)	0.224
Active society	1.04	(0.93–1.17)	0.52
Active environment	1.00	(0.92–1.09)	0.96
Active people	0.93	(0.84–1.02)	0.133
National diabetes policies and registers	0.98	(0.86–1.11)	0.705
Sex (ref: Males)			
Females	0.77	(0.75–0.80)	<0.001
Age groups (ref: Age group 15–44)			
45–64	4.95	(4.65–5.27)	<0.001
65 and above	8.74	(8.17–9.34)	<0.001
Degree of urbanization (ref: Cities)			
Town and suburbs	0.91	(0.87–0.94)	<0.001
Rural areas	0.85	(0.82–0.88)	<0.001
Educational attainment (ref: Primary/less than primary education)			
Secondary education	0.76	(0.73–0.79)	<0.001
High education	0.65	(0.62–0.69)	<0.001
Labor status (ref: employed)			
Unemployed	1.32	(1.22–1.43)	<0.001
Others	1.91	(1.82–2.00)	<0.001
Net monthly equalized income of the household (ref: between 1st quintile and 2nd quintile)			
Between 2nd quintile and 4th quintile	0.90	(0.87–0.94)	<0.001
Between 4th quintile and 5th quintile	0.78	(0.74–0.82)	<0.001
BMI (kg/m^2^) (Ref: <25)			
25–29.9	1.74	(1.67–1.81)	<0.001
≥30	3.76	(3.61–3.92)	<0.001
Frequency of eating fruits (ref: one and more per day)			
1 to 6 times a week	0.90	(0.87–0.94)	<0.001
Less than once a week and never	0.93	(0.87–1.00)	0.051
Frequency of eating vegetables (ref: one and more per day)			
1 to 6 times a week	0.99	(0.96–1.03)	0.606
Less than once a week and never	1.01	(0.93–1.10)	0.745

Legend: BMI body mass index (kg/m^2^), ICC = intraclass correlation.

## Data Availability

Policy data are available in public databases, World Cancer Research Fund International’s (WCRFINT) NOURISHING and MOVING policy databases https://policydatabase.wcrf.org/, accessed on 3 March 2021, the European Coalition for Diabetes report “Diabetes in Europe policy puzzle: the state we are in” https://www.fend.org/wp-content/uploads/2021/04/Policy-Puzzle-PP4finalweb.pdf, accessed on 2 January 2021. Microdata of EHIS 2014 is available via Eurostat upon request.

## Supplementary Materials

The following are available online at https://www.mdpi.com/article/10.3390/nu13103439/s1, Table S1: Number of implemented government policy actions promoting healthy diets and targeting physical activity, national diabetes plans in effect and national diabetes registers in operation in the EU member states, 2014. Table S2: Definitions of the variables (EHIS), Table S3: Distribution of demographic, socioeconomic and lifestyle characteristics of the study population in 2014.
