# Supplementary material for: Impact of Policies in Nutrition and Physical Activity on Diabetes and Its Risk Factors in the 28 Member States of the European Union
Source: Nutrients. 2021 Sep 28;13(10):3439. doi: 10.3390/nu13103439 (PMC8537865; doi:10.3390/nu13103439)
Supplement: Supplementary file 1 [file nutrients-13-03439-s001.zip › nutrients-1287055-supplementary.pdf]

Authors: Szabolcs Lovas[1], Nour Mahrouseh[1], Olaniyan Bolaji[2], Carlos Alexandre Soares Andrade[1], Diana Wangeshi Njuguna[1], Noel Nellamkuzhi[2] and Orsolya Varga[1]

[1]Department of Public Health and Epidemiology, Faculty of Medicine, University of Debrecen, 26 Kassai Street, 4028 Debrecen, Hungary
[2]Faculty of Medicine, University of Debrecen, Debrecen, Hungary

Supplementary file S1:

Table S1. Number of implemented government policy actions promoting healthy diets and targeting physical activity, national diabetes plans in effect and national diabetes register in operation in the EU member states, 2014

	Type	Categories	Policy Areas	Sub-policy Areas	Austria	Belgium	Bulgaria	Czech Republic	Denmark	Germany	Estonia	Ireland	Greece	Spain	France	Croatia	Italy	Cyprus	Latvia	Lithuania	Luxembourg	Hungary	Malta	Netherlands	Poland	Portugal	Romania	Slovenia	Slovakia	Finland	Sweden	United Kingdom
Policies	Nutritional	Food Environment	Nutrition label standards and regulations on the use of claims and implied claims on food	Mandatory nutrient lists on packaged food*	0	0	0	0	0	0	0	0	0	0	0	0	0	0	0	0	0	0	0	0	0	0	0	0	0	0	0	1
				Trans fats included in mandatory nutrient labels	0	0	0	0	0	0	0	0	0	0	0	0	0	0	0	0	0	0	0	0	0	0	0	0	0	0	0	0
				Clearly visible "interpretative" labels and warning labels	0	1	0	1	2	0	0	0	0	0	0	0	0	0	0	1	0	0	0	1	1	0	0	1	0	1	1	2
				On-shelf labelling	0	0	0	0	0	0	0	0	0	0	0	0	0	0	0	0	0	0	0	0	0	0	0	0	0	0	0	0
				Calorie and nutrient labelling on menus and displays in out-of-home venues	0	0	0	0	0	0	0	0	0	0	0	0	0	0	0	0	0	0	0	0	0	0	0	0	0	0	0	1
				Warning labels on menus and displays in out-of-home venues	0	0	0	0	0	0	0	0	0	0	0	0	0	0	0	0	0	0	0	0	0	0	0	0	0	0	0	0
				Rules on nutrient claims (ie nutrient content and nutrient comparative claims)	1	1	1	1	1	1	1	1	1	1	1	1	1	1	1	1	1	0	1	1	1	1	1	1	1	1	1	1
				Rules on health claims (ie nutrient function and disease risk reduction claims)	1	1	1	1	1	1	1	0	1	1	1	1	1	1	0	0	1	1	1	1	1	1	1	1	1	1	1	1
			Offer healthy food and set standards in public institutions and other specific settings	Fruit & vegetable initiatives in schools	0	0	0	0	0	0	0	0	0	0	0	0	0	0	0	0	0	0	0	0	0	0	0	0	0	0	0	0
				Mandatory standards for food available in schools including restrictions on unhealthy food	0	0	0	0	0	0	0	0	0	0	0	0	0	0	0	0	0	0	0	0	0	0	0	0	0	0	0	0
				Mandatory standards for food available in schools and in their immediate vicinity	0	0	0	0	0	0	0	0	0	0	0	0	0	0	0	0	0	0	0	0	0	0	0	0	0	0	0	0
				Voluntary guidelines for food available in schools	0	0	0	0	0	0	0	0	0	0	0	0	0	0	0	0	0	0	0	0	0	0	0	0	0	0	0	0
				Bans specific to vending machines in schools	0	0	0	0	0	0	0	0	0	0	0	0	0	0	0	0	0	0	0	0	0	0	0	0	0	0	0	0
				Standards in social support programmes	0	0	0	0	0	0	0	0	0	0	0	0	0	0	0	0	0	0	0	0	0	0	0	0	0	0	0	0
				Standards in other specific locations (eg health facilities workplace)	0	0	0	0	0	1	0	0	0	0	0	0	0	0	1	0	0	0	0	0	0	0	0	0	0	1	0	2
			Use economic tools to address food affordability and purchase incentives	Health-related food taxes	0	0	0	0	0	0	0	0	0	0	1	0	0	0	1	0	0	1	0	0	0	0	0	0	0	0	0	0
				Voluntary health-related food taxes	0	0	0	0	0	0	0	0	0	0	0	0	0	0	0	0	0	0	0	0	0	0	0	0	0	0	0	0
				Increasing import tariffs on specified "unhealthy" food	0	0	0	0	0	0	0	0	0	0	0	0	0	0	0	0	0	0	0	0	0	0	0	0	0	0	0	0
				Lowering import tariffs on specified "healthy" food	0	0	0	0	0	0	0	0	0	0	0	0	0	0	0	0	0	0	0	0	0	0	0	0	0	0	0	0

				Targeted subsidies for healthy food*	0	0	0	0	0	0	0	0	0	0	0	0	0	0	0	0	0	0	0	0	0	0	0	0	0	0	0	0
			Restrict food advertising and other forms of commercial promotion	Mandatory regulation of broadcast food advertising to children	0	0	0	0	0	0	0	0	0	0	0	0	0	0	0	0	0	0	0	0	0	0	0	0	0	0	0	0
				Mandatory regulation of food advertising on non-broadcast communications channels	0	0	0	0	0	0	0	0	0	0	0	0	0	0	0	0	0	0	0	0	0	0	0	0	0	0	0	0
				Mandatory regulation of food advertising through any medium	0	0	0	0	0	0	0	0	0	0	0	0	0	0	0	0	0	0	0	0	0	0	0	0	0	0	0	0
				Mandatory regulation of specific marketing techniques	0	0	0	0	0	0	0	0	0	0	0	0	0	0	0	0	0	0	0	0	0	0	0	0	0	0	0	0
				Mandatory regulation of marketing of specific food items and beverages	0	0	0	0	0	0	0	0	0	0	0	0	0	0	0	0	0	0	0	0	0	0	0	0	0	0	0	0
				Mandatory regulation of food marketing in schools	0	0	0	0	0	0	0	0	0	0	0	0	0	0	0	0	0	0	0	0	0	0	0	0	0	0	0	0
				Mandatory requirement that advertisements must carry a health message or warning	0	0	0	0	0	0	0	0	0	0	1	0	0	0	0	0	0	0	0	0	0	0	0	0	0	0	0	0
				Voluntary regulation of food advertising on non-broadcast communications channels	0	0	0	0	0	0	0	0	0	0	0	0	0	0	0	0	0	0	0	0	0	0	0	0	0	0	0	0
				Government engage with industry to develop self-regulation to restrict food marketing to children	0	0	0	0	0	0	0	0	0	0	0	0	0	0	0	0	0	0	0	0	0	0	0	0	0	0	0	0
				Government support voluntary pledges developed by industry	0	1	0	0	0	0	0	0	0	0	0	0	0	0	0	0	0	0	0	0	0	0	0	0	0	0	0	0
			Improve nutritional quality of the whole food supply	Voluntary reformulation of food products	1	0	0	1	0	0	0	1	0	1	1	1	1	0	0	0	0	1	0	1	0	0	0	0	0	0	0	1
				Voluntary commitments to reduce portion sizes	0	0	0	0	0	0	0	0	0	0	0	0	0	0	0	0	0	0	0	0	0	0	0	0	0	0	0	1
				Mandatory limits on level of salt in food products	0	1	1	0	0	0	0	0	1	0	0	0	0	0	0	0	0	1	0	1	0	1	0	0	0	0	0	0
				Mandatory removal of trans fats in food products	1	0	0	0	1	0	0	0	0	0	0	0	0	0	0	0	0	1	0	0	0	0	0	0	0	0	0	0
				Limits on the availability of high-fat meat products	0	0	0	0	0	0	0	0	0	0	0	0	0	0	0	0	0	0	0	0	0	0	0	0	0	0	0	0
				Limits on the availability of high-sugar food products and beverages	0	0	0	0	0	0	0	0	0	0	0	0	0	0	0	0	0	0	0	0	0	0	0	0	0	0	0	0
			Set incentives and rules to create a healthy retail and food service environment	Incentives and rules for stores to locate in under-served neighbourhoods	0	0	0	0	0	0	0	0	0	0	0	0	0	0	0	0	0	0	0	0	0	0	0	0	0	0	0	0
				Initiatives to increase the availability of healthier food in stores and food service outlets	0	0	0	0	0	0	0	0	0	0	0	0	0	0	0	0	0	0	0	0	0	0	0	0	0	0	0	1
				Incentives and rules to reduce trans fat in food service outlets	0	0	0	0	0	0	0	0	0	0	0	0	0	0	0	0	0	0	0	0	0	0	0	0	0	0	0	0
				Incentives and rules to offer healthy food options as a default in food service outlets	0	0	0	0	0	0	0	0	0	0	0	0	0	0	0	0	0	0	0	0	0	0	0	0	0	0	0	0
				Incentives and rules to restrict sugar-sweetened beverage consumption	0	0	0	0	0	0	0	0	0	0	0	0	0	0	0	0	0	0	0	0	0	0	0	0	0	0	0	0
				Incentives and rules to reduce salt in food service outlets	0	0	0	0	0	0	0	0	0	0	0	0	0	0	0	0	0	0	0	0	0	0	0	0	0	0	0	0
				Planning restrictions on food outlets	0	0	0	0	0	0	0	0	0	0	0	0	0	0	0	0	0	0	0	0	0	0	0	0	0	0	0	1
		Food System	Harness supply chain and actions across sectors to ensure coherence with health	Working with food suppliers to provide healthier ingredients	0	0	0	0	0	0	0	0	0	0	0	0	0	0	0	0	0	0	0	0	0	0	0	0	0	0	0	1
				Nutrition standards for public procurement	0	0	0	0	0	0	0	0	0	0	0	0	0	0	0	0	0	0	0	0	0	0	0	0	0	0	0	1
				Public procurement through "short" chains (eg local farmers)	0	0	0	0	0	0	0	0	0	0	0	0	0	0	0	0	0	0	0	0	0	0	0	0	0	0	0	0
				Supply chain incentives for food production	0	0	0	0	0	0	0	0	0	0	0	0	0	0	0	0	0	0	0	0	0	0	0	0	0	0	0	0

				Supporting urban agriculture in health and planning policies	0	0	0	0	0	0	0	0	0	0	0	0	0	0	0	0	0	0	0	0	0	0	0	0	0	0	0	0
				Community food production	0	0	0	0	0	0	0	0	0	0	0	0	0	0	0	0	0	0	0	0	0	0	0	0	0	0	0	0
				Governance structures for multi-sectoral/stakeholder engagement	0	0	0	0	0	0	0	0	0	0	0	0	0	0	0	0	0	0	0	0	0	0	0	0	0	1	0	0
		Behaviour Change Communication	Inform people about food and nutrition through public awareness	Development and communication of food-based dietary guidelines	1	0	1	0	0	1	0	0	1	0	1	1	0	1	1	0	0	1	0	0	1	1	1	1	0	0	0	0
				Development and communication of guidelines for specific food groups	0	0	0	0	0	0	0	0	0	0	0	0	0	0	0	0	0	0	0	0	0	0	0	0	0	1	0	0
				Public awareness, mass media and informational campaigns and social marketing on healthy eating	0	0	0	0	0	0	0	0	0	0	1	0	0	0	0	0	0	0	0	1	0	0	0	0	0	0	0	1
				Public awareness campaigns specific to fruit and vegetables	0	0	0	0	1	0	0	0	0	1	1	0	0	0	0	0	0	0	0	0	0	0	0	0	0	0	0	0
				Public awareness campaigns concerning specific unhealthy food and beverages*	0	0	0	0	0	0	0	0	0	0	0	0	0	0	0	0	0	0	0	0	0	0	0	0	0	0	0	0
				Public awareness campaigns concerning salt	0	0	0	0	0	0	1	0	0	0	0	0	0	0	0	0	0	0	0	0	0	0	0	0	0	0	0	1
			Nutrition advice and counselling in healthcare settings	Guidelines and programmes to provide support in primary care to people who are overweight and obese	0	0	0	0	0	0	0	0	0	1	0	0	0	0	0	0	0	0	0	0	0	0	0	0	0	0	0	1
				Nutrition counselling in primary care	0	0	0	0	0	0	0	0	0	0	0	0	0	0	0	0	0	0	0	0	0	0	0	0	0	0	0	0
				Training for health professionals	0	0	0	0	0	1	0	0	0	0	0	0	0	0	0	0	0	0	0	0	0	0	0	0	0	0	0	2
			Give nutrition education and skills	Nutrition education on curricula	0	0	0	0	0	0	0	0	0	0	0	0	0	0	0	0	0	0	0	0	0	0	0	0	0	0	0	0
				Community-based nutrition education	0	0	0	0	0	0	0	0	0	0	0	0	0	0	0	0	0	0	0	0	0	0	0	0	0	0	0	0
				Cooking skills	0	0	0	0	0	0	0	0	0	0	0	0	0	0	0	0	0	0	0	0	0	0	0	0	0	0	0	0
				Initiatives to train school children on growing food	0	0	0	0	0	0	0	0	0	0	0	0	0	0	0	0	0	0	0	0	0	0	0	0	0	0	0	0
				Workplace or community health schemes	0	0	0	0	0	0	0	0	0	0	0	0	0	0	0	0	0	0	0	0	0	0	0	0	0	0	0	0
				Training for caterers and food service providers	0	0	0	0	0	0	0	0	0	0	0	0	0	0	0	0	0	0	0	0	0	0	0	0	0	0	0	0
	Physical	Active Societies	Make opportunities and initiatives that promote physical activity in schools, the community and sport and recreation	Physical education in the curriculum	0	0	0	0	0	0	0	0	0	0	0	0	0	0	1	0	0	0	0	0	0	0	0	0	0	0	0	0
				Policies increasing physical activity in and outside of classrooms	0	0	0	0	0	0	0	0	0	0	0	0	0	0	0	0	0	0	0	0	0	0	0	0	0	0	0	0
				Community initiatives promoting physical activity across the life course	0	0	0	0	0	0	0	0	0	0	0	0	1	0	0	0	0	0	0	0	0	0	0	0	0	0	0	0
				Mass participation initiatives promoting physical activity across the life course	0	0	0	0	0	0	0	0	0	0	0	0	0	0	0	0	0	0	0	0	0	0	0	0	0	0	0	0
				Policies promoting/supporting physical activity for least active groups and vulnerable/marginalised people	0	0	0	0	0	1	0	0	0	0	0	0	0	0	0	0	0	0	0	0	0	2	0	0	0	0	0	0
				Policies promoting/supporting physical activity for people of all ages and abilities	0	0	0	0	0	0	0	0	0	0	0	0	0	0	0	0	0	0	0	0	1	1	0	0	0	0	0	2
				Financial and non-financial incentives to promote physical activity	0	0	0	0	0	0	0	0	0	0	0	0	0	0	0	0	0	0	0	1	1	0	0	0	0	0	0	0
			Offer physical activity opportunities in the workplace and training in physical activity promotion across multiple professions	Pre-and in-service training for sport educators/trainers on inclusive sport	0	0	0	0	0	0	0	0	0	1	0	0	0	0	0	0	0	0	0	0	0	1	0	0	0	0	0	0
				Pre-and in-service training for relevant professions outside of health care (such as educators, architects, planners, landscape architects, park and recreation professionals)	0	0	0	0	0	1	0	0	0	0	2	0	0	0	0	0	0	0	0	0	0	0	0	0	0	0	0	0

		Policies promoting physical activity in the workplace	0	0	0	0	0	0	0	0	0	1	0	0	0	0	0	0	0	0	0	0	0	0	0	0	0	0	0	1
Active Environments	Visualise and enact structures and surroundings which promote physical activity	Design guidelines and regulations for buildings that prioritise equitable, safe, and universal access by all, that encourage occupants to be physically active	0	0	0	0	0	0	0	0	0	0	1	0	0	0	0	0	0	0	0	0	0	1	0	0	0	0	0	0
		Active design guidelines outside buildings	0	0	0	0	0	0	0	0	0	0	0	0	0	0	0	0	0	0	0	0	0	0	0	0	0	0	0	0
		Active design guidelines for open/green spaces	0	0	0	0	0	0	0	0	0	0	0	0	0	0	0	0	0	0	0	0	0	0	0	0	0	0	0	0
		Walking and cycling infrastructure	0	0	0	0	0	2	0	0	0	1	2	0	0	0	1	0	0	0	0	1	0	1	0	0	0	0	0	2
		Sport facilities infrastructure	0	0	0	0	0	0	0	0	0	0	0	0	0	0	0	0	0	0	0	0	0	0	0	0	0	0	0	0
		Policies that support access to quality public open space and green spaces	0	0	0	0	0	0	0	0	0	0	0	0	0	0	0	0	0	0	0	0	0	0	0	0	0	0	0	0
		Policies that support people of all ages and abilities considered and accounted for in all planning decisions	0	0	0	0	0	0	0	0	0	0	0	0	0	0	0	0	0	0	0	0	0	0	0	0	0	0	0	0
	Implement transport infrastructure and opportunities that support active societies	Policies that support public transport	0	0	0	0	0	0	0	0	0	0	1	0	0	0	0	0	0	0	0	0	0	0	0	0	0	0	0	0
		Road safety actions including safety of pedestrians, cyclists etc	0	0	0	0	0	2	0	0	0	0	0	0	0	0	0	0	0	0	0	0	0	1	0	0	0	0	0	1
		Mass communication campaigns to increase awareness about road safety	0	0	0	0	0	1	0	0	0	0	0	0	0	0	0	0	0	0	0	0	0	0	0	0	0	0	0	0
		Mass communication campaigns to promote the use of public transport	0	0	0	0	0	0	0	0	0	0	0	0	0	0	0	0	0	0	0	0	0	0	0	0	0	0	0	0
		Mass communication campaigns to promote active transport	0	0	0	0	0	1	0	0	0	0	0	0	0	0	0	0	0	0	0	0	0	0	0	0	0	0	0	1
		Policies promoting active transport	0	0	0	0	0	1	0	0	0	0	0	0	0	0	0	0	0	0	0	1	0	0	0	0	0	0	0	0
		Policies promoting active transport to and from school	0	0	0	0	0	0	0	0	0	0	0	0	0	0	0	0	0	0	0	0	0	0	0	0	0	0	0	0
		Policies promoting active transport to and from work	0	0	0	0	0	0	0	0	0	0	0	0	0	0	0	0	0	0	0	0	0	0	0	0	0	0	0	1
Active People	Normalise and increase physical activity through public communication that motivates and builds behaviour change skills	Mass communication campaigns including social marketing to increase awareness and knowledge about benefits of physical activity through the life course	0	0	0	0	0	0	0	0	0	3	0	0	1	0	0	0	0	0	0	0	0	0	0	0	0	0	0	0
		Mass communication campaigns including social marketing to change social norms about the accessibility and need for physical activity	0	0	0	0	0	0	0	0	0	0	0	0	0	0	0	0	0	0	0	0	0	0	0	0	0	0	0	2
		Mass communication campaigns to increase awareness of co-benefits of physical activity (environment, social, and economic) through the life course	0	0	0	0	0	0	0	0	0	0	0	0	0	0	0	0	0	0	0	0	0	0	0	0	0	0	0	0
		Mass communication campaigns to change social norms about discrimination and gender equality in sport	0	0	0	0	0	0	0	0	0	1	0	0	0	0	0	0	0	0	0	0	0	0	0	0	0	0	0	0
		Develop and communicate physical activity guidelines	0	0	0	0	0	0	0	0	0	0	1	0	0	0	0	0	0	0	0	0	1	0	0	0	0	0	0	1
	Give physical activity training, assessment and counselling in healthcare settings	Pre- and in-service training within health care	0	0	0	0	0	0	0	0	0	1	0	0	0	0	0	0	0	0	0	0	0	0	0	0	0	0	0	0
		Primary care (assessment, counselling and physical activity prescriptions)	0	0	0	0	0	0	0	0	0	0	0	0	0	0	0	0	0	0	0	1	0	0	0	0	0	0	0	4
		Policies that promote physical activity in health care and outpatient settings	0	0	0	0	0	0	0	0	0	0	0	0	0	0	0	0	0	0	0	0	0	0	0	0	0	0	0	0
		Policies that promote physical activity in social provision	0	0	0	0	0	0	0	0	0	0	0	0	0	0	0	0	0	0	0	0	0	0	0	0	0	0	0	0
National Diabetes Plan			Yes	No	Yes	Yes	Yes	No	No	Yes	Yes	Yes	No	Yes	Yes	No	No	No	No	No	No	Yes	Yes	Yes	Yes	Yes	Yes	Yes	Yes	No

**Registeries**		No	No	No	No	Yes	No	No	No	No	No	No	Yes	No	Yes	Yes	No	No	No	Yes	No	No	Yes	No	No	No	No	Yes	No
**Diabetes Burden**	Prevalence	4.93	5.34	6.37	7.67	4.62	7.17	5.49	4.63	9.24	6.83	9.98	7.13	6.66	6.06	4.66	4.41	5.57	8.06	8.25	5.38	6.64	9.33	4.79	6.85	6.86	7.73	4.75	5.8

Table S2: Definitions of the variables based on the European Health Interview Survey 2014 (EHIS wave 2)

Sex was derived from the question “Sex”. The self-reported answers were split into two categories, male and female.
Age was derived from the question “Age of respondent in completed years at the time of the interview”. The self-reported answers were split into three categories (15-44, 45-64, 65 and above).
Degree of urbanization was derived from the question “Degree of urbanisation”. The self-reported answers were split into three categories. Cities: derived from self-reported answers (densely-populated area) in 2014. Towns and suburbs: derived from self-reported answers (intermediate-populated area) in 2014, and self-reported answers (Towns and suburbs) in 2019. Rural areas: derived from self-reported answers (thinly-populated area).
Educational level was derived from the question “What is the highest education leaving certificate, diploma or education degree you have obtained?”. The self-reported answers were split into three categories. Primary or less than primary education: derived from self-reported answers (no formal education or below, primary education, lower secondary education) in 2014. Secondary education: derived from self-reported answers (upper secondary education, post-secondary but non-tertiary education tertiary education; short-cycle). Higher education: derived from self-reported answers (bachelor level or equivalent, master level or equivalent, doctoral level or equivalent).
Labour status was derived from the question “How would you define your current labour status?”. The self-reported answers were split into three categories. Employed: derived from self-reported answers (carries out a job or profession, including unpaid work for a family business or holding, an apprenticeship or paid traineeship, etc.). Unemployed: derived from the self-reported answer (unemployed). Others:, self-reported answers (pupil, student, further training, unpaid work experience, In retirement or early retirement or has given up business, permanently disabled, in compulsory military or community service, fulfilling domestic tasks and other inactive person).
Net monthly equivalised income of the household the respondent belong to was derived from the question”” The self-reported answers were split into three categories. Between 1st quintile and 2nd quintile: derived from the self-reported answers (Below 1st quintile, Between 1st quintile and 2nd quintile). Between 2nd quintile and 4th quintile: derived from self-reported answers (Between 2nd quintile and 3rd quintile and Between 3rd quintile and 4th quintile). Between 4th quintile and 5th quintile: derived from self-report answers (Between 4th quintile and 5th quintile).
BMI was calculated derived from two questions (How tall are you without shoes?) and (How much do you weigh without clothes and shoes?). The self-reported answers were available in centimeters (cm) and kilograms(kg), respectively, calculated derived from the formula “kg/m^2” and split into three categories (<25 , 25-29.9 and ≥30).
Frequency of eating fruits was based the question “How often do you eat fruits (excluding juice)?”. The self-reported answers were split into three categories. One and more per day derived from self-reported answers (once or more a day). 1 to 6 times a week derived from self-reported answers (less than once a day but at least 4 times a week and less than 4 times a week and but at least once a week). Less than once a week and never: derived from the self-reported answers (less than once a week and never).

Frequency of eating vegetables or salad was based the question “How often do you eat vegetables or salad (excluding juice and potatoes)?”. The self-reported answers were split into three categories. One and more per day derived from self-reported answers (once or more a day). 1 to 6 times a week derived from self-reported answers (less than once a day but at least 4 times a week, less than 4 times a week and but at least once a week). Less than once a week and never: derived from the self-reported answers (less than once a week and Never).

Source: European Health Interview Survey (EHIS wave 2) Methodological manual: 2013 edition
